# Colon capsule endoscopy investigation based on faecal haemoglobin concentration in symptomatic patients to detect bowel disease

**DOI:** 10.1093/bjsopen/zrae007

**Published:** 2024-02-13

**Authors:** Campbell MacLeod, Craig Mowat, Jemma Hudson, Judith Strachan, Angus James Mackintosh Watson

**Affiliations:** Department of Surgery, Aberdeen Royal Infirmary, Aberdeen, UK; Institute of Applied Health Sciences, University of Aberdeen, Aberdeen, UK; Population Health & Genomics, School of Medicine, Ninewells Hospital and Medical School, University of Dundee, Dundee, UK; Health Services Research Unit, University of Aberdeen, Aberdeen, UK; Department of Blood Sciences, Ninewells Hospital and Medical School, Dundee, UK; Department of Surgery, Raigmore Hospital, Inverness, UK

## Introduction

The faecal immunochemical test (FIT), which measures the concentration of faecal haemoglobin (f-Hb), is now advocated by the Association of Coloproctology of Great Britain and Ireland and the British Society of Gastroenterology for use in primary care as an adjunct to clinical acumen in the assessment of new bowel symptoms^1^. FIT is superior to symptoms at predicting the presence of significant bowel disease (SBD)^2–4^. Higher f-Hb concentrations correspond with an increased risk of harbouring colorectal pathology and as such can be used to prioritize patients for investigation.

The COVID-19 pandemic has significantly increased the waiting times for diagnostic tests due to pauses in service and reduced capacity^5^. To generate diagnostic capacity, colon capsule endoscopy (CCE), a non-invasive alternative to colonoscopy, has been implemented in the UK^6^. To date, there is no published evidence reporting the clinical outcomes for symptomatic patients undergoing CCE based on their f-Hb. The aim of this study, using data from the ScotCap evaluation, was to examine the need for further test following CCE and yield of SBD at CCE and at follow-up endoscopy according to the patient’s FIT result at the point of referral.

## Methods

This was a subgroup analysis of patients who participated in the ScotCap evaluation, a multicentre, prospective clinical evaluation of CCE^7^. Symptomatic (patients with new symptoms suggestive of colorectal pathology, see Supplementary materials for referral symptoms) NHS Highland patients who underwent CCE between June 2019 and May 2020, and had a FIT carried out in primary care, were included in the study. Further details on the patient recruitment, bowel preparation and booster regimen used, full inclusion and exclusion criteria, data collection and analysis are described elsewhere^7^. The protocol for this study is available online^8^.

For this analysis, patients who were investigated by CCE were separated into categories based on their f-Hb (<10, 10–399, ≥ 400 µg/g). SBD was defined as a colorectal cancer (CRC), higher risk adenoma (HRA, defined as any polyp ≥10 mm) and inflammatory bowel disease. The presence of 3 or more polyps was not included in the HRA definition given the potential for CCE to double report polyps due to the nature of the test. The primary outcome was to measure the need for further test at different f-Hb cut-offs. Data were analysed using SPSS (version 27, IBM). Further details on methods (FIT testing, outcomes and statistical analysis) and description of results can be found in the Supplementary materials. This work was a subgroup analysis of a published clinical evaluation in which patients provided written consent to take part in, and did not require ethical approval.

## Results

Over the 9-month study period, of the 509 patients who underwent CCE, 316 patients were in the symptomatic cohort and of those 316 patients, 203 (64%) had an available FIT result; thus, they were included in this analysis (*Fig. S1*). *Table 1* shows the demographic characteristics and patient outcomes according to f-Hb range. The proportion of patients who had a FIT result in the f-Hb ranges <10 µg/g, 10–399 µg/g, and ≥400 µg/g was 55.7%, 41.9% and 2.5%, respectively. In the 10–399 µg/g range, the median f-Hb in the cohort was 29 µg/g and 72 of the 85 patients (84.7%) had an f-Hb <100 µg/g. Patients with an f-Hb <10 µg/g were more likely to require no further test following CCE than those with an f-Hb 10–399 µg/g (43.4% *versus* 24.7%, *P* = 0.007). *Table 2* shows CCE and follow-up test findings according to f-Hb range. SBD was reported by CCE in 21.2% and 37.7% (*P* = 0.011) of patients with an f-Hb <10 µg/g and 10–399 µg/g, respectively. Subsequent follow-up endoscopy test found 8.5% and 21% of patients had SBD in the f-Hb <10 µg/g and 10–399 µg/g cohorts, respectively.

**Table 1:** Demographic and patient outcomes by faecal haemoglobin (f-Hb) range

f-Hb range (µg/g)	Total *n* = 203	<10 *n* = 113	10–399 *n* = 85	*P*	≥400* n = 5
Age (years), median(s.d.)	61(11.9)	59(11.9)	64(12.3)	0.184	66(4.4)
**Sex**					
Female	119 (58.6)	62 (54.9)	54 (63.5)	0.221	3 (60.0)
Male	84 (41.4)	51 (45.1)	31 (36.5)		2 (40.0)
Hb (g/l), mean(s.d.), *n*	140.9(11.6); 160	141.6(10.3); 92	140(13.3); 64	0.402	139.5(11.9); 4
**Referral urgency**					
Urgent suspected cancer	84 (41.4)	46 (40.7)	34 (40.0)	0.370	4 (80.0)
Urgent	64 (31.5)	32 (28.3)	31 (36.5)		1 (20.0)
Routine	55 (27.1)	35 (31.0)	20 (23.5)		0 (0.0)
Complete test rate†	146 (71.9)	82 (72.6)	60 (70.6)	0.760	4 (80.0)
Adequate preparation rate‡	162 (79.8)	91 (80.5)	67 (78.8)	0.767	4 (80.0)
Successful test rate§	134 (66.0)	75 (66.4)	55 (64.7)	0.807	4 (80.0)
**Outcomes**					
No further test	73 (36.0)	49 (43.4)	21 (24.7)	0.007	3 (60.0)
Colonoscopy	67 (33.0)	33 (29.2)	32 (37.6)	0.210	2 (40.0)
Due to CCE findings	61 (91.0)	31 (93.9)	28 (87.5)		2 (100)
Inadequate CCE	6 (9.0)	2 (6.1)	4 (12.5)		0 (0)
Flexible sigmoidoscopy	56 (27.6)	26 (23.0)	30 (35.3)	0.057	0 (0.0)
Due to CCE findings	21 (37.5)	8 (30.8)	13 (43.3)		0 (0.0)
Inadequate CCE	35 (62.5)	18 (69.2)	17 (56.7)		0 (0.0)
CT colonogram	4 (2.0)	4 (3.5)	0 (0.0)	0.136	0 (0.0)
Other¶	3 (1.5)	1 (0.9)	2 (2.4)	0.578	0 (0.0)

Values are *n* (column per cent) unless otherwise stated. *The ≥400 µg/g cohort was excluded from statistical analysis. †The proportion of patients whose whole colon and rectum were visualized. ‡The proportion of patients with bowel preparation rated at least fair in all segments of the colon and assessed as acceptable overall by the CCE reader. §The proportion of patients with a complete test and adequate bowel preparation. ¶Patients requiring review or laparotomy.

**Table 2:** CCE and follow up test findings by f-Hb range

f-Hb range (µg/g)	Total *n* = 203	<10 *n* = 113	10–399 *n* = 85	*P*	≥400* n = 5
**Number of polyps reported by CCE**					
0	52 (25.6)	28 (24.8)	21 (24.7)	0.996	3 (60.0)
1–5	106 (52.2)	60 (53.1)	44 (51.8)		2 (40.0)
6–9	36 (17.1)	20 (17.7)	16 (18.8)		0 (0.0)
≥10	9 (4.4)	5 (4.4)	4 (4.7)		0 (0.0)
**Largest size of polyp found at CCE, *n* (% of those with polyp reported)**					
<6 mm	45 (29.8)	30 (35.3)	14 (21.9)	0.095	1 (50.0)
6–9 mm	57 (37.7)	33 (38.8)	24 (37.5)		0 (0.0)
≥10 mm	49 (32.5)	22 (25.9)	26 (40.6)		1 (50.0)
Colonic inflammation reported by CCE	9 (4.4)	2 (1.8)	6 (7.1)	0.061	1 (20.0)
Colorectal cancer reported by CCE	0 (0.0)	0 (0.0)	0 (0.0)		0 (0.0)
Significant bowel disease reported by CCE†	58 (28.6)	24 (21.2)	32 (37.7)	0.011	2 (40.0)
Number of patients undergoing colonoscopy or flexible sigmoidoscopy, *n*	123 (60.6)	59 (52.2)	62 (72.9)		2 (40.0)
**Number of polyps found at colonoscopy or flexible sigmoidoscopy**					
0	63 (51.2)	31 (52.5)	30 (48.4)	0.259	2 (100.0)
1–5	50 (40.7)	26 (44.1)	24 (38.7)		0 (0.0)
6–9	9 (7.3)	2 (3.4)	7 (11.3)		0 (0.0)
≥10	1 (0.8)	0 (0.0)	1 (1.6)		0 (0.0)
**Largest size of polyp at colonoscopy or flexible sigmoidoscopy, *n* (% of those with polyp found)**					
<6 mm	27 (45.0)	17 (60.7)	10 (31.3)	0.065	0 (0.0)
6–9 mm	20 (33.3)	6 (21.4)	14 (43.8)		0 (0.0)
≥10 mm	13 (21.7)	5 (17.9)	8 (25.0)		0 (0.0)
Colonic inflammation found at colonoscopy or flexible sigmoidoscopy	6 (4.9)	0 (0.0)	5 (8.1)	0.023	1 (50.0)
Colorectal cancer found at colonoscopy or flexible sigmoidoscopy	0 (0.0)	0 (0.0)	0 (0.0)		0 (0.0)
Significant bowel disease found at colonoscopy or flexible sigmoidoscopy†	19 (15.4)	5 (8.5)	13 (21.0)		1 (50.0)

Values are *n* (per cent) unless otherwise stated. *The ≥400 µg/g cohort was excluded from statistical analysis. †Significant bowel disease defined as high-risk adenoma (≥10 mm), inflammatory bowel disease or colorectal cancer.

## Discussion

In this subgroup analysis of the ScotCap evaluation, the outcomes for symptomatic patients triaged to CCE were reported, where vetting secondary care clinicians can use FIT results to influence decision-making. The majority of patients triaged to CCE had an f-Hb <10 µg/g. Patients with an f-Hb <10 µg/g were less likely to require a follow-up test after CCE. However, a significant proportion of patients with FIT <10 µg/g had SBD diagnosed by CCE (21.2%). Similar rates of SBD have been reported for patients with an f-Hb <10 µg/g in FIT diagnostic accuracy studies^4,9^. However, SBD was only seen in 8.5% of patients undergoing colonoscopy/flexible sigmoidoscopy, after CCE in the <10 µg/g group. There are three possible explanations for the discrepancy. First, CCE may be more sensitive at detecting mucosal lesions compared to colonoscopy. Second, the size of polyps detected by CCE may be overestimated by up to 30%^10^. Finally, CCE can record the same polyp more than once, thereby reporting the same polyp as 2 or more polyps, and subsequent colonoscopy only finds a single polyp. If, however, any of these scenarios are true, up to 1 in 10 patients with a FIT <10 µg/g have significant bowel disease.

The optimum f-Hb threshold for choosing to investigate patients with CCE, rather than colonoscopy, must be balanced against the likely proportion of patients who will require further investigation if pathology is found. A wide variation in risk of underlying SBD exists in the group of patients with an f-Hb between 10 and 399 µg/g^11^. The benefits of CCE will be lost if the f-Hb threshold for CCE is too high due to an increased follow-up test rate. Too low, and excess patients will be triaged to colonoscopy, lowering the yield of pathology and subsequently raising the number of patients required to undergo colonoscopy to diagnose CRC (number needed to scope, NNS). Differing approaches have been taken in the UK with CCE being offered to patients with an f-Hb between 10 and 100 µg/g in NHS England and between 10 and 150 µg/g in NHS Scotland. The ongoing evaluation of CCE in these countries should produce data sets to allow the optimum FIT threshold for CCE to be calculated. Given the high rate of SBD in those with an f-Hb ≥400 µg/g, we would advocate that these patients are offered colonoscopy in the first instance.

It has not been possible to provide any guidance on the combined use of FIT and CCE to diagnose CRC in this work because no patients were diagnosed with CRC by CCE, or subsequent follow-up test, in the ScotCap evaluation. The lack of CRC diagnosed in this study may be a reflection of clinicians selecting patients with a low risk of harbouring CRC who are likely to be best suited to undergoing CCE. However, the importance of non-cancer diagnoses should not be overlooked, as most symptomatic patients undergoing lower GI investigation (even in 2-week wait or urgent suspect cancer pathways) will not have CRC^12,13^. These results indicate that CCE is likely to be well placed to investigate lower-risk patients who may have benign pathology such as polyps and suspected inflammatory bowel disease who can subsequently be triaged for targeted endoscopy. The patients with no significant pathology will avoid colonoscopy (36% of all symptomatic patients undergoing CCE) and can be safely discharged with macroscopic colonic disease excluded.

In this study, the patients selected for CCE were found to have an f-Hb in the lower concentration ranges, their corresponding yield of SBD was low, and many required no further test. This would indicate a possible niche for CCE to occupy and thereby ease the burden on endoscopy units. Further work should focus on establishing the optimum FIT range for patients undergoing CCE.

## Supplementary Material

zrae007_Supplementary_Data

## Data Availability

The data sets generated during and analysed during the current study are not publicly available due to the clinical nature of the data, but are available from the corresponding author on reasonable request.
