# Supplementary material for: Colon capsule endoscopy investigation based on faecal haemoglobin concentration in symptomatic patients to detect bowel disease
Source: BJS Open. 2024 Feb 13;8(1):zrae007. doi: 10.1093/bjsopen/zrae007 (PMC10869214; doi:10.1093/bjsopen/zrae007)

# Colon capsule endoscopy investigation based on faecal haemoglobin concentration in symptomatic patients to detect bowel disease

C MacLeod (0000-0001-9161-0661)^1,2^, C Mowat (0000-0002-5021-415X)^3^, J Hudson^4^, J Strachan (0000-0003-4528-1005)^5^, AJM Watson^6^ on behalf of the ScotCap clinical leads

^1^ Department of Surgery, Aberdeen Royal Infirmary, Aberdeen, UK

^2^ Institute of Applied Health Sciences, University of Aberdeen, Aberdeen, UK

^3^ Population Health & Genomics, School of Medicine, Ninewells Hospital and Medical School, University of Dundee, Dundee, UK

^4^ Health Services Research Unit, University of Aberdeen, Health Sciences Building, Aberdeen, UK

^5^ Department of Blood Sciences, Ninewells Hospital and Medical School, Dundee, UK

^6^ Department of Surgery, Raigmore Hospital, Inverness, UK

**Corresponding author**

Mr Campbell MacLeod

Email – [Campbell.macleod@nhs.scot](mailto:Campbell.macleod@nhs.scot)

Address – Department of Surgery, Aberdeen Royal Infirmary, Foresterhill Road, Aberdeen, AB25 2ZN, UK

ORCID- 0000-0001-9161-0661

**Supplementary Materials - Index**

| **Supplementary Table 1** |  |
| --- | --- |
| Patients referral symptoms | *Page 2* |
| **Supplementary Text 1** |  |
| Methods and results | *Page 3* |
| **Supplementary Figure 1** |  |
| Study flow | *Page 6* |

# Supplementary Table 1. Patients’ referral symptoms

| **Referral symptoms** | | | | |
| --- | --- | --- | --- | --- |
| **f-Hb range (µg/g)** | **Total**  **N=203** | **<10**  **N=113** | **10-399**  **N=85** | **≥400**  **N=5** |
| Referral symptoms^1^ |  |  |  |  |
| Change in bowel habit | 136 (67.0) | 85 (75.2) | 50 (58.8) | 1 (20.0) |
| Abdominal Pain | 82 (40.4) | 50 (44.2) | 31 (36.5) | 1 (20.0) |
| Rectal bleeding | 35 (17.2) | 16 (14.2) | 17 (20.0) | 2 (40.0) |
| Positive FIT | 56 (27.6) | 0 (0.0) | 52 (61.2) | 4 (80.0) |
| Diarrhoea | 34 (16.7) | 20 (17.7) | 13 (15.3) | 1 (20.0) |
| Constipation | 27 (13.3) | 12 (10.6) | 14 (16.5) | 1 (20.0) |
| Weight loss | 19 (9.4) | 10 (8.8) | 9 (10.6) | 0 (0.0) |
| Microcytic anaemia | 6 (3.0) | 2 (1.8) | 4 (4.7) | 0 (0.0) |
| Other | 8 (3.9) | 7 (6.2) | 1 (1.2) | 0 (0.0) |
| *Values are N (percent).*^1^*More than 1 symptom could be chosen.* | | | | |

# Supplementary Text 1. Methods and results

# **Method**

**Study design and population**

This was a subgroup analysis of patients who participated in the ScotCap evaluation; a multicentre, prospective clinical evaluation of CCE(11). Patients were referred from primary care with lower GI symptoms via established colorectal referral pathways. FIT was being used by primary care clinicians as an adjunct to clinical acumen. Patients were triaged to CCE (as opposed to colonoscopy/CT colonoscopy/sigmoidoscopy/face to face clinic) by the vetting consultant colorectal surgeon based on the referral information provided. Symptomatic patients who underwent CCE between June 2019 and May 2020 with an available FIT result were included in the study. Of the health boards included in the evaluation (NHS Highland, Western Isles and Grampian), only patients from NHS Highland were included based on the availability of FIT results. Patients excluded from the CCE evaluation were those with slow transit constipation (unlikely to expel the capsule) or diarrhoea (high need for mucosal biopsies) and microcytic anaemia as their sole reason for investigation (likely to require bidirectional endoscopy). Patients underwent CCE supervised by a trained nurse following a standard protocol. The bowel preparation and booster regimen that was used is described in Supplementary Materials. Further details on the patient recruitment, full inclusion and exclusion criteria, data collection and analysis are described elsewhere(11). The protocol for this study is available online(https://osf.io/nrxdq/files/osfstorage/61dd6b5dff8fbd0119840609).

For the purpose of this analysis, only symptomatic patients, defined as patients with symptoms suggestive of colorectal pathology requiring investigation, were retrospectively selected from the original cohort.

**Faecal immunochemical test collection and analysis**

FIT has been available for use in primary care in NHS Highland since December 2018. GPs were given guidance on the use of FIT when the test was introduced to the health board and were strongly encouraged to carry out a FIT prior to referral to secondary care for patients with symptoms suggestive of colorectal cancer or SBD.

During the study period, patients were supplied with a FIT by their GP following initial consultation. The FIT was accompanied by a pictorial instruction sheet and return envelope. The specimen collection device (EXTEL HEMO-AUTO MC Collection Picker, Minaris Medical CO., Ltd, supplied by Alpha Labs Ltd) collects a 2mg sample of stool which is inserted into a vial containing 2ml of buffer. Tests were returned to GP practices and transported at ambient temperature to Blood Sciences, Ninewells Hospital, Dundee using routine sample transport services. On arrival samples were stored at 4°C until analysed using an HM-JACKarc analyser(Minaris Medical Co.,Ltd) with a limit of detection of 2µg/g, limit of quantitation of 7µg/g and upper measurement limit of 400µg/g. Results were returned to the requesting GP and uploaded to the patient’s electronic health record.

Patients who were investigated by CCE were separated into categories based on their f-Hb (<10, 10-399, ≥400µg/g) for analysis. These values were chosen based on the reported risk of SBD at these thresholds in previously published studies(4,13). A cut-off of 10µg/g was used to define a positive test as per the National Institute for Health and Care Excellence (NICE) DG30 and, ACPGBI and BSG guidance(1,14). A complete test was defined as a CCE which visualised the whole colon and rectum. The bowel preparation, scored according to the Boston Bowel Preparation Scale, was considered adequate if rated at least fair in all colonic segments, and the overall quality was deemed adequate by the CCE reader(15). A CCE test was successful if it was complete and the bowel preparation was adequate. Follow up tests were classified as “due to CCE findings” if there were any findings reported by CCE necessitating endoscopy, regardless of whether the CCE examination was adequate. Investigations carried out solely due to an incomplete CCE and or inadequate bowel preparation were classified as “inadequate procedure”. The presence of 3 or more polyps was not included in the HRA definition given the potential for CCE to double report polyps due to the nature of the test. In addition, the presence of high-grade dysplasia and villous features were not included in the definition of HRA since the focus of this study was the rate of SBD as detected by CCE.

**Outcomes of interest**

The primary outcome was to measure the need for further test at different f-Hb cut-offs. Secondary outcomes included calculating the proportion of patients with SBD, the number and size of polyps detected by CCE and follow up test, and CCE test performance (completion, adequate bowel preparation and successful test rate) at different f-Hb thresholds.

**Statistics**

Continuous variables such as age and Hb count were summarised using median and mean values, respectively. The <10µg/g and 10-399µg/g f-Hb cohorts were compared using the chi-squared test and Fishers exact for categorical variables, and analysis of variance for continuous variables. The ≥400µg/g f-Hb cohort was excluded from statistical analysis due to the comparatively small sample size. The ≥400µg/g cohort was not combined with the 10-399µg/g since it would likely distort any outcomes for the latter group due to the significantly higher risk of pathology in patients with a f-Hb ≥400µg/g. A p-value of <0.05 was considered significant. Data was analysed using SPSS (version 27, IBM).

# **Results**

Over the 9-month study period, of the 733 patients who were invited to take part in the ScotCap evaluation, 509 patients underwent CCE of which 316 patients were in the symptomatic cohort. Of those 316 patents, 203 (64%) had an available FIT result and were included in this analysis (Figure 1). The remaining 113 (36%) patients did not have a FIT carried out. The median time between the FIT being reported and the GP referral to secondary care was 8 days. Table 1 shows the demographic characteristics and patient outcomes according to f-Hb range. The median age was 61 years and 58.6% were female. The proportion of patients who had a FIT result in the f-Hb ranges <10µg/g, 10-399µg/g, and ≥400µg/g was 55.7%, 41.9% and 2.5%, respectively. In the 10-399µg/g range, the median f-Hb in the cohort was 29µg/g and 72 of the 85 patients (84.7%) had a f-Hb <100µg/g. The mean Hb was 140.9g/l for the 160/203 (78.8%) patients who had a Hb result accompanying referral. CCE test performance (completion rate, adequate bowel preparation and successful test rate) was consistent across cohorts. The rate of complete test, adequate bowel preparation and successful test for all patients was 71.9%, 79.8% and 66%, respectively.

Patients with a f-Hb <10µg/g were significantly more likely to require no further test compared to those with a f-Hb between 10-399µg/g (43.3% vs 24.7%, *p*=0.007). Table 2 shows CCE findings according to f-Hb range, and Table 3 shows follow up test findings according to f-Hb range. SBD was identified in 58 patients (28.6%). Of the 123 (60.6%) attending follow-up endoscopy (colonoscopy or flexible sigmoidoscopy), only 19 patients (15.4%) were confirmed to have SBD. Patients in the 10-399µg/g cohort were more likely to have SBD detected by CCE compared to the <10µg/g cohort (37.7% vs 21.2% *p*=0.011). Similarly, colonic inflammation was more likely to be detected at colonoscopy or flexible sigmoidoscopy in the 10-399µg/g group than the <10µg/g group (8.1% vs 0% *p*=0.023).

Supplementary Figure 1. Study flow


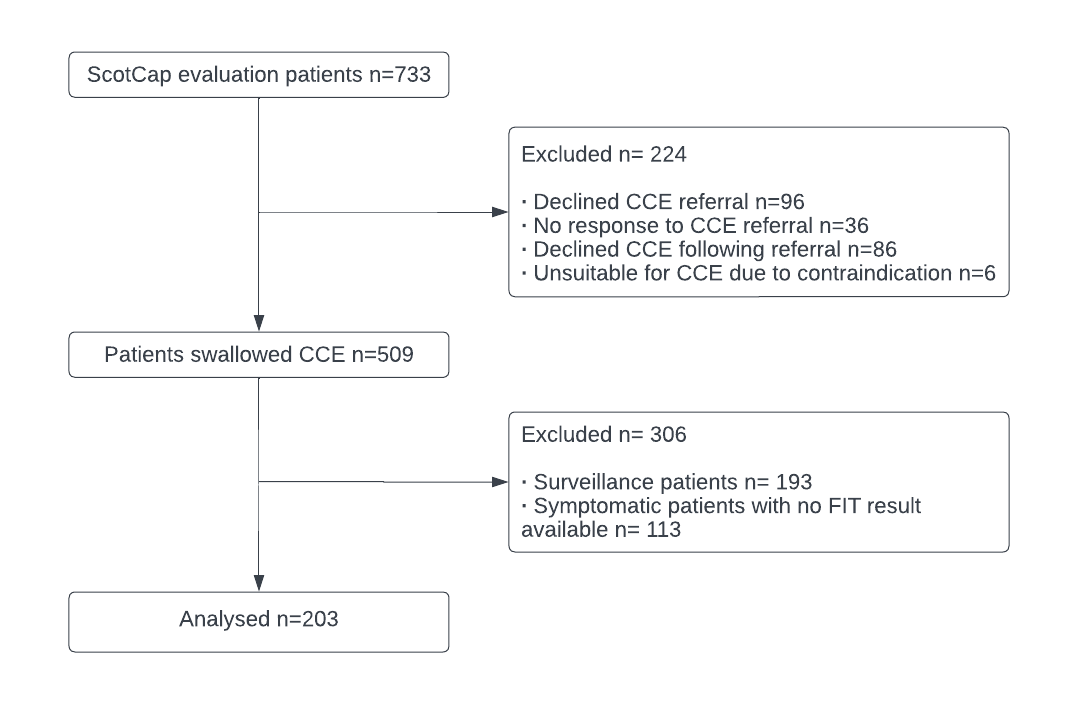

Supplement: zrae007_Supplementary_Data [file zrae007_supplementary_data.docx]
